# Lactic Dehydrogenase to Albumin Ratio Is Associated With the Risk of Stroke-Associated Pneumonia in Patients With Acute Ischemic Stroke

**DOI:** 10.3389/fnut.2021.743216

**Published:** 2021-09-16

**Authors:** Dan Yan, Qiqi Huang, Caijun Dai, Wenwei Ren, Siyan Chen

**Affiliations:** ^1^Department of Pulmonary and Critical Care Medicine, Jinhua Municipal Central Hospital, The Affiliated Jinhua Hospital, College of Medicine, Zhejiang University, Jinhua, China; ^2^Faculty of Nursing, Burapha University, Saen Suk, Thailand; ^3^Department of Neurology, The First Affiliated Hospital of Wenzhou Medical University, Wenzhou, China

**Keywords:** stroke, pneumonia, lactic dehydrogenase, albumin, ratio

## Abstract

**Background:** Stroke-associated pneumonia (SAP) is one of the common complications of stroke patients. Higher lactic dehydrogenase (LDH) and lower albumin levels were associated with SAP, but the contribution of the LDH to albumin ratio (LAR) to the risk of SAP in acute ischemic stroke (AIS) patients remained unclear.

**Methods:** A total of 3173 AIS patients were included in this study, divided into SAP (*n* = 417) and non-SAP groups (*n* = 2756). Characteristics were compared between these two groups. The receiver operating characteristic curves (ROC) were used to evaluate the discrimination ability of the LAR, LDH, and albumin levels in predicting SAP. Logistic regression analysis was furtherly adopted to estimate the association between LAR and SAP. We also used the restricted cubic spline (RCS) to clarify the relationship between LAR and the risk of SAP.

**Results:** LAR in the SAP group was significantly higher than that of the non-SAP group (8.75 ± 4.58 vs. 6.10 ± 2.55, *P* < 0.001). According to the results of ROC, LAR had the highest prognostic accuracy compared to LDH and albumin (*P* < 0.05). Besides, the logistic regression model showed that higher LAR (LAR > 6.75) were more vulnerable to SAP (OR, 2.80; 95% CI, 2.18–3.59, *P* < 0.001), controlling the confounders. The RCS model showed that there was a non-linear relationship between LAR and the risk of SAP.

**Conclusion:** High LAR was associated with an increased risk of SAP in patients with AIS. LAR may be a potential predictor for the incidence of SAP. Appropriate prevention measures were needed in patients with high LAR (LAR > 6.75).

## Introduction

Acute ischemic stroke (AIS) caused by the interruption of blood flow to specific areas of the brain is a common type of stroke with high mortality, accounting for approximately 85% of acute stroke patients (1). These patients occasionally present with a variety of symptoms (such as hemiparesis, vertigo, ataxia, sensory deficits, and dysphagia) and complications such as pneumonia, deep vein thrombosis, and urinary tract infections (1). Pneumonia is one of the most frequent complications after stroke affecting 14% of patients and may lead to prolonged hospitalization, increased financial burden, higher morbidity and mortality of stroke patients (2, 3). The pathophysiology of SAP is multi-factorial. Stroke-induced confusion, dysphagia, and immune deficiency, nasogastric tube feeding, aspiration, and poor oral hygiene can increase the risk of pneumonia (4). Early identification and intervention of stroke-associated pneumonia (SAP) would be of high value and had aroused great attention in these years.

LDH and albumin are two non-specific inflammatory biomarkers (5). Albumin synthesized by hepatocytes has been promoted as a highly sensitive marker of the nutritional status in clinical practice (6). Hypoproteinemia is often discovered in nutritional deterioration and inflammation-related disease (7). Previous studies showed a high level of albumin might be linked to decreased risk of SAP, and had prognostic value in predicting the risk of pneumonia in AIS patients (8). Another study indicated that albumin was a highly sensitive biomarker in predicting the prognosis of community-acquired pneumonia (9). A recent study also indicated that albumin may be a predictor for the severity of COVID-19 (10). LDH is a cytoplasmic enzyme that exists in different tissues and it is an indicator of cell destruction caused by inflammation or other pathological conditions (11). The relationship between LDH and SAP had not been explored before. However, LDH was found to be related to pneumonia in other populations. For example, a recent study suggested that LDH could be identified as an important predictor of acute respiratory distresssyndrome (ARDS) and used to predict the mortality of ARDS patients (12). Besides, higher LDH levels were also found to be associated with the poor prognosis of COVID-19 (13–15). Another study showed that LDH might be a helpful marker to predict the incidence of postoperative pneumonia in patients with aneurysmal subarachnoid hemorrhage (16). Considering the role of LDH and albumin in pneumonia, it would be interesting to explore whether a new indicator combing LDH and albumin would show higher value in predicting SAP.

In recent studies, LDH to albumin ratio (LAR) was found to be related to lung diseases, including pulmonary thromboembolism (17) and community-acquired pneumonia (9). However, the role of LAR in predicting the incidence of pneumonia in AIS patients had not been investigated. Based on these findings, we sought to identify whether LAR also played a role in SAP. We assumed that higher LAR was associated with a higher risk of SAP in AIS patients.

## Methods

### Study Population

**Figure 1:**
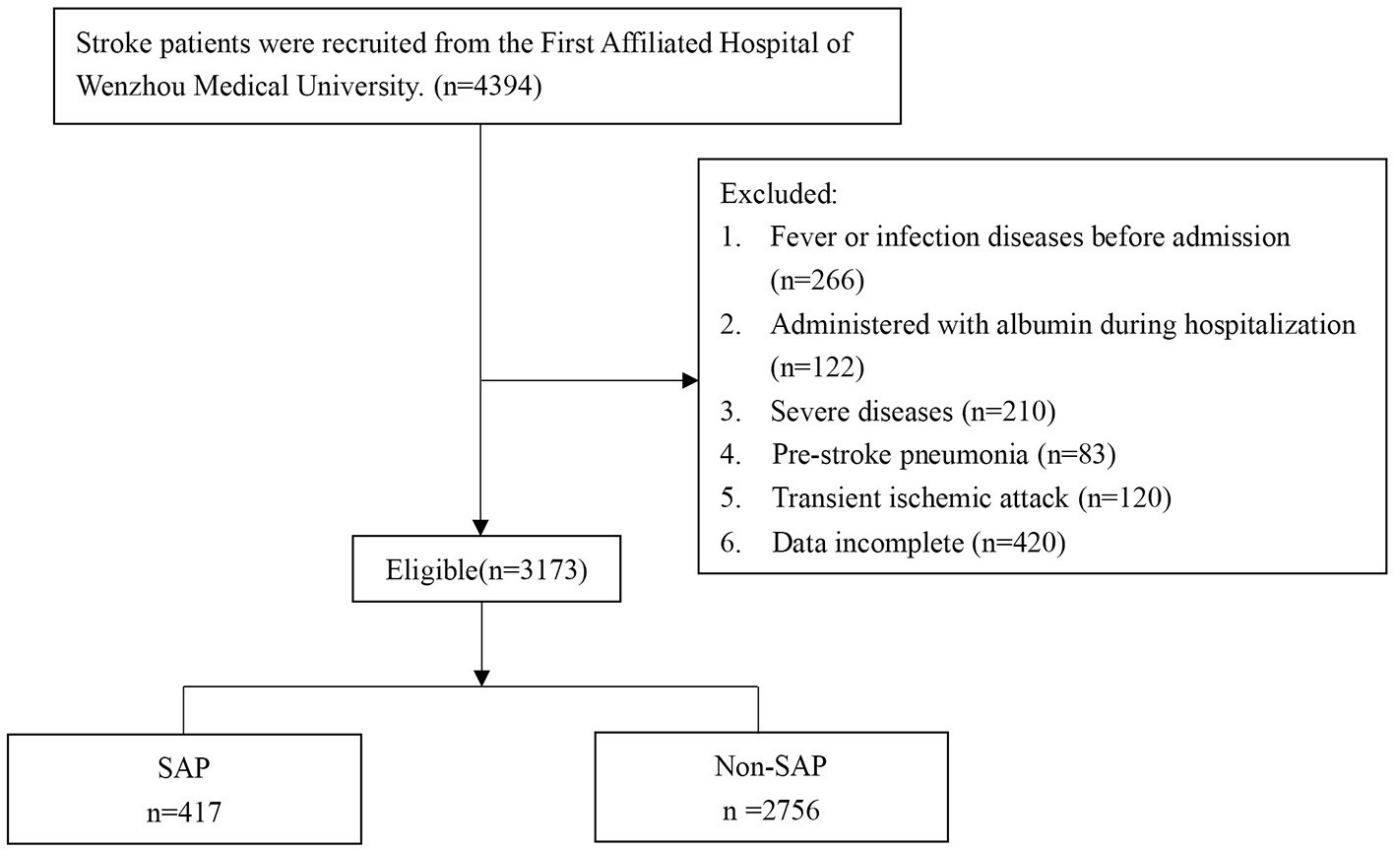
Flow of participants. SAP, stroke-associated pneumonia.

This was a retrospective study, and patients were recruited from the First Affiliated Hospital of Wenzhou Medical University between February 2013 and March 2021 (Figure 1). The inclusion criteria were as follows: (1) AIS confirmed by magnetic resonance imaging (MRI) or cranial computed tomography (CT) at the outpatient clinic before hospitalization; (2) ≥18 years old; (3) stroke onset within one week.

The exclusion criteria were as follows: (1) fever or infectious diseases within two weeks before admission; (2) severe diseases including cancer, heart failure, renal insufficiency, acute myocardial infarction or liver dysfunction; (3) pneumonia before stroke; (4) transient ischemic attack; (5) administered with albumin during hospitalization; (6) incomplete information.

As shown in Figure 1, a total of 4,394 patients were screened, and 3,173 eligible patients were eventually recruited and divided into SAP group (*n* = 417) and Non-SAP group (*n* = 2756).

The study was approved by the Ethics Committee of the First Affiliated Hospital of Wenzhou Medical University. All patient data included in the study was anonymous, so informed consent could be exempted (18).

### Data Collection

We obtained clinical features and demographic characteristics from self-reported electronic medical records at the time of hospital admission, which included age, gender, body mass index (BMI), hypertension, diabetes, liver disease, atrial fibrillation, smoking and drinking history, duration of hospitalization, National Institutes of Health Stroke Scale (NIHSS) score, stroke classification (TOAST criteria), dysphagia, and laboratory parameters (albumin, leukocyte, erythrocyte, LDH, hemoglobin, and LAR). SAP was diagnosed according to the modified Centers for Disease Control and Prevention criteria of hospital-acquired pneumonia (19) during the first 7 days after admission to the hospital, combing the clinical, radiological, and laboratory parameters of respiratory tract infection (20).

The National Institutes of Health Stroke Scale (NIHSS) score and dysphagia were evaluated by trained neurologists within 24 hours after admission. NIHSS score was used to assess stroke severity: 0–4, mild; 5–15, moderate; and ≥16, severe. According to the results of the dysphagia assessment, patients were managed with different diets (ordinary diets, semi-liquid diet, viscous paste meal, and nasal feeding). The LAR was calculated by dividing the LDH concentration by the albumin concentration. On the second day after admission, fasting blood samples were collected at 8:00–10:00 to measure biochemical indicators.

### Statistical Analyses

Descriptive statistics were used to summarize baseline characteristics. Continuous variables were expressed as median (interquartile) and mean (standard deviation). Categorical variables were evaluated by calculating frequencies or percentages. Univariate analyses were conducted to analyze differences between two groups comparison were analyzed by Mann-Whitney U test, *t*-test (continuous variable), and chi-square test or the Fisher's exact test (categorical variable). The receiver operating characteristic (ROC) curves were used to evaluate the discrimination ability of LAR, LDH, and albumin levels in predicting SAP. Multivariable logistic regression analysis was adopted to estimate the association between LAR and SAP, and the results were visualized as a forest plot. Significant variables (*P* < 0.05) in the univariate analysis were considered as confounders and were included in the multivariable logistic regression analysis. We also used the restricted cubic spline (RCS) to clarify the relationship between LAR and the risk of SAP. The spline was defined using 4 knots at prespecified locations according to the percentiles of the distribution of LAR, 5th, 35th, 65th, and 95th percentiles. All statistical analyses were conducted by using R v3.5.1 with *P* < 0.05 as the significance threshold.

## Results

### Baseline Characteristics

A total of 3,173 AIS patients (1,970 males and 1,203 females) were included in this study, of which SAP patients accounted for 13.14% (417/3,173), and non-SAP 86.86% (2,756/3,173). The LAR of the SAP group was significantly higher than that of the non-SAP group (8.75 ± 4.58 vs. 6.10 ± 2.55, *P* < 0.001). Patients in the SAP group were elder and presented longer hospitalizations, more severe dysphagia, higher NIHSS score, lower albumin, and higher levels of leukocytes, as well as LDH. Furthermore, the SAP group had a higher proportion of patients with atrial fibrillation. All the differences mentioned above were statistically significant (*P* < 0.05). The proportion of large artery atherosclerosis, hypertension, diabetes, kidney disease, liver disease, smoking, and drinking history did not differ between SAP and non-SAP groups (Table 1).

**Table 1:** Comparisons of clinical characteristics between SAP and Non-SAP groups.

**Variables**	**Total(*n* = 3,173)**	**Non-SAP(*n* = 2,756)**	**SAP(*n* = 417)**	***P***
**Demographic characteristic**				
Age	67.97 ± 11.63	67.39 ± 11.51	71.77 ± 11.69	<0.001
Gender (%)				0.091
Male	1970 (62.09)	1695 (61.5)	275 (65.95)	
Female	1203 (37.91)	1061 (38.5)	142 (34.05)	
BMI (kg/m^2^)	24.71 ± 18.29	24.50 ± 15.45	26.12 ± 31.10	0.939
**Clinical parameters**				
Dysphagia (%)				<0.001
Ordinary diet	877 (27.64)	839 (30.44)	38 (9.11)	
Semi-liquid diet	1417 (44.66)	1323 (48)	94 (22.54)	
Viscous paste meal	100 (3.15)	84 (3.05)	16 (3.84)	
Nasal feeding	779 (24.55)	510 (18.51)	269 (64.51)	
TOAST (%)				0.559
Large artery atherosclerosis	786 (24.77)	688 (24.96)	98 (23.5)	
Other	2387 (75.23)	2068 (75.04)	319 (76.5)	
NIHSS, median (IQR)	4 (2, 9)	4 (2, 8)	10 (5, 15)	<0.001
NIHSS (%)				<0.001
0-4	1657 (52.22)	1565 (56.79)	92 (22.06)	
5-15	1295 (40.81)	1067 (38.72)	228 (54.68)	
≥16	221 (6.97)	124 (4.5)	97 (23.26)	
Duration of hospitalization	12.45 ± 7.60	11.93 ± 6.88	15.93 ± 10.66	<0.001
Hypertension (%)	2196 (69.21)	1914 (69.45)	282 (67.63)	0.487
Diabetes (%)	853 (26.88)	739 (26.81)	114 (27.34)	0.868
Kidney disease (%)	59 (1.86)	46 (1.67)	13 (3.12)	0.065
Liver disease (%)	58 (1.83)	53 (1.92)	5 (1.20)	0.405
Atrial fibrillation (%)	604 (19.04)	450 (16.33)	154 (36.93)	<0.001
Drinking history (%)	940 (29.62)	823 (29.86)	117 (28.06)	0.487
Smoking history (%)	1133 (35.71)	989 (35.89)	144 (34.53)	0.629
**Laboratory parameters**				
Albumin(g/L)	37.24 ± 4.02	37.49 ± 3.81	35.58 ± 4.88	<0.001
LDH(U/L)	235.63 ± 93.99	225.58 ± 81.62	302.07 ± 134.71	<0.001
Leukocyte(10^9^/L)	7.73 ± 2.75	7.51 ± 2.55	9.14 ± 3.52	<0.001
Erythrocyte(10^9^/L)	4.41 ± 0.55	4.42 ± 0.54	4.37 ± 0.64	0.077
Hemoglobin(g/dL)	134.40 ± 17.01	134.66 ± 16.70	132.91 ± 18.88	0.080
LAR	6.45 ± 3.04	6.10 ± 2.55	8.75 ± 4.58	<0.001

*LDH, lactic dehydrogenase; BMI, body mass index; SAP, stroke-associated pneumonia; LAR, LDH to albumin ratio*.

### The Predictive Value of LAR in SAP

**Figure 2:**
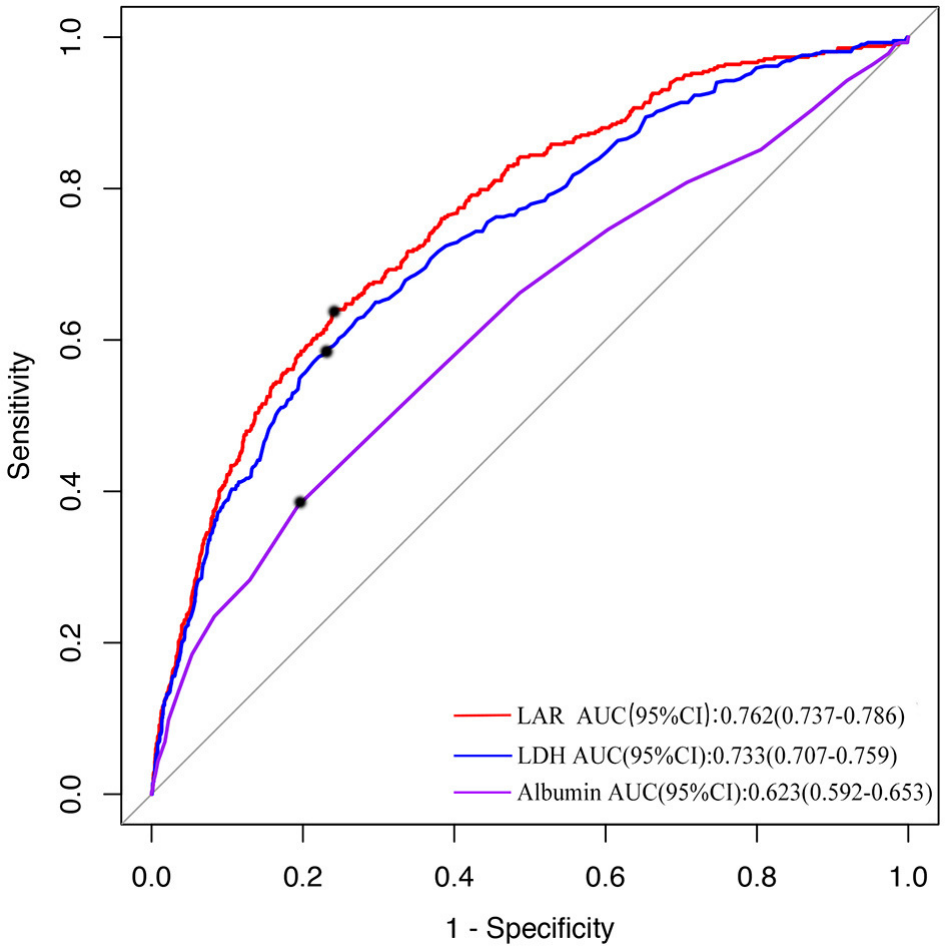
The ROC curve of LAR in predicting the incidence of SAP. The area under the ROC curve (AUC) shows the predictive ability of LAR. The AUC was 0.762 (95% CI 0.737–0.786) of LAR in predicting the risk of SAP. LAR, Lactic dehydrogenase to albumin ratio; AUC, Area under the curve; SAP, stroke-associated pneumonia; ROC, The receiver operating characteristic.

As was shown in Figure 2, the area under the ROC curve (AUC) for the predictive model which was used to assess the discriminatory ability of LAR in predicting the incidence of SAP in AIS patients was 0.762 (95% CI 0.737–0.786). With a cut-off value of 6.75, the prediction of SAP was the best as follows: 74.2% accuracy, 75.9% specificity, and 63.8% sensitivity. Additionally, the AUC of LDH was 0.733 (95% CI 0.707–0.759) with 77.1% specificity and 58.8% sensitivity. The AUC of albumin was 0.623 (95% CI 0.592–0.653) with 80.6% specificity and 38.1% sensitivity. LAR showed had a higher prognostic accuracy compared to LDH and albumin (*p* < 0.05).

### Multivariable Logistic Regression Analysis of the Associations Between LAR and SAP

**Figure 3:**
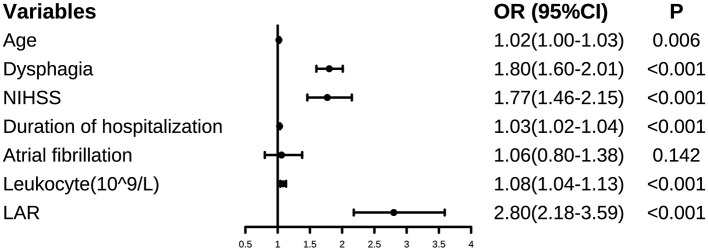
Forest plot of odds ratios (OR) for SAP. An OR>1 meant an increased risk of SAP, and an OR<1 meant the opposite.

Based on the best cut-off value of ROC, patients were divided into high-LAR (LAR > 6.75) and low-LAR groups (LAR ≤ 6.75). The following logistic regression analysis showed that the high-LAR group (LAR > 6.75) was more susceptible to the development of SAP (OR: 5.54, 95%CI: 4.46–6.90, *P* < 0.001). As was shown in Figure 3, after adjusting for the confounders (Age, NIHSS score, dysphagia, length of hospitalization, atrial fibrillation, and leukocyte), the findings remained unchanged (OR: 2.80, 95%CI: 2.18–3.59, *P* < 0.001).

In addition, when LAR was included in the regression model as a continuous variable, the results remained significant in both unadjusted (OR: 1.70, 95%CI: 1.57–1.84, *P* < 0.001) and adjusted model (OR: 1.32, 95%CI: 1.21–1.44, *P* < 0.001). An OR > 1 meant LAR was a risk factor for SAP and an OR <1 meant the opposite.

### The Non-linear Relationship Between LAR and the Risk of SAP

**Figure 4:**
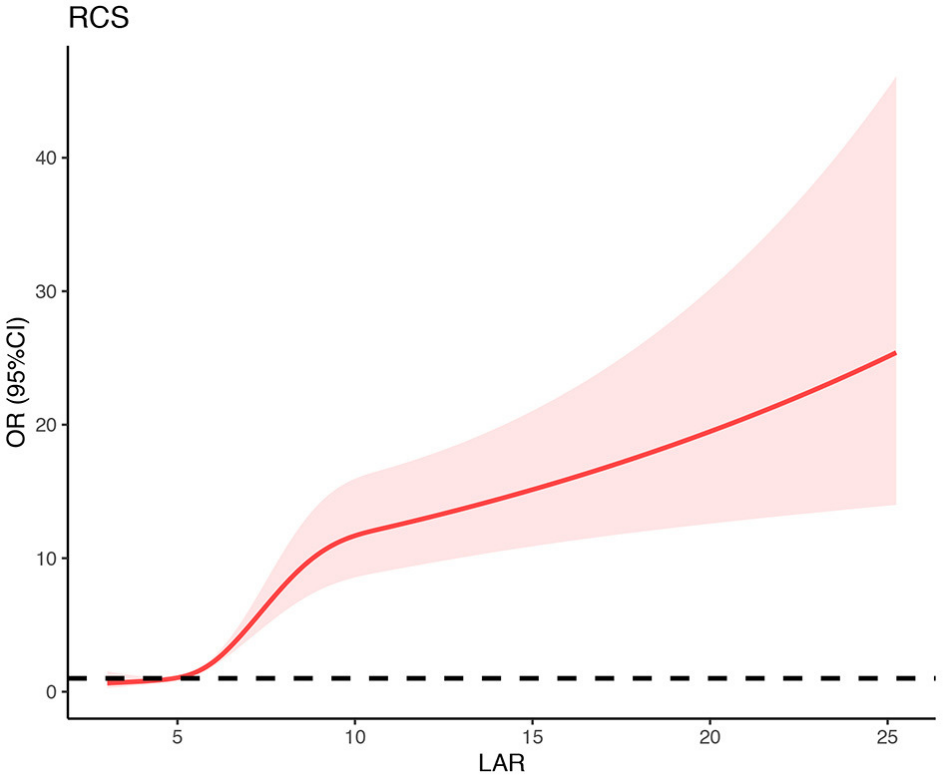
Association between LAR and risk of SAP using restricted cubic spline (RCS) analysis. Risk of developing SAP in AIS patients increased with advanced LAR. When the value of LAR ranged from 6–10, the risk of SAP increased rapidly with the elevation of LAR. While, the risk of SAP was relatively stable, with the value of LAR <6 or >10. SAP, stroke-associated pneumonia; OR, odds ratio; 95%CI, 95% confidence interval.

As was shown in Figure 4, the RCS model indicated that there was a non-linear association between LAR and the risk of SAP (P for non-linearity < 0.001). When the value of LAR ranged from 6–10, the risk of SAP increased rapidly with the elevation of LAR (Figure 4). While, the risk of SAP was relatively stable, with the value of LAR <6 or >10.

## Discussion

To our knowledge, this was the first study investigating the association between LAR and SAP. The main results of this study were as follows: (1) LAR was strongly associated with the incidence of SAP. Patients with higher LAR (>6.75) had a nearly three-fold higher risk of SAP. (2)There was a non-linear relationship between LAR and the incidence of SAP. (3) LAR might be a potential predictor for SAP in AIS patients.

LDH and albumin are both easily obtained in routine clinical practice, and both of them were related to pneumonia. Our study indicated that LAR combining LDH and albumin was associated with the risk of pneumonia in AIS patients. Patients with higher LAR (>6.75) had a nearly three-fold higher risk of SAP. Furthermore, the risk of SAP increased rapidly with the elevation of LAR when the value of LAR ranged from 6–10; while the rising trend of SAP development was relatively flat with the value of LAR <6 or > 10. There are several possible explanations for the close relationship between the LAR and SAP. The first explanation may be that both albumin and LDH can affect the immune system. Low albumin levels can lead to poor nutritional reserves and a weak immune system, which may increase the risk of pneumonia (21). Besides, LDH is not only a metabolite but also a prognostic biomarker for immune surveillance (22). Specifically, LDH can increase the production of lactic acid, leading to the inhibition of cytolytic cells (such as cytotoxic T-lymphocytes and natural killer cells) and enhancement of immune-suppressive cells (such as dendritic cells and tumor-associated macrophages) (22). The second explanation may be owing to the neuroprotective effect of high albumin and low LDH levels, which may indirectly influence the risk of SAP by improving the severity of stroke. Albumin is an important antioxidant that may exert neuroprotective effects on stroke through antioxidant activity and anti-inflammatory activity (23). Besides, albumin can reverse blood stagnation and thrombosis, which can help maintain the vascular patency after successful thrombolysis (24, 25). Moreover, the inhibitory effect of albumin on the hemoglobin concentration and erythrocyte sedimentation rate may promote reperfusion (26). Researchers also found that the inhibition of LDH was considered to have a neuroprotective effect in cerebral ischemic stroke (27). The third explanation may be that both of them can modulate the inflammatory reaction. Albumin can influence endotoxin-induced inflammation via binding of the lipid A portion, reactive oxygen species, lipopolysaccharide, and other bacterial products (lipoteichoic acid and peptidoglycan) (28, 29). Additionally, albumin expands the volume of plasma, which may be related to its role in regulating systemic inflammation (29). LDH played a crucial role in inflammation through promoting T cell effector functions (30). Besides, LDH can strengthen the inflammatory response caused by lipopolysaccharide in macrophages (31). However, further studies were needed to clarify the specific mechanism.

It is known that LDH and albumin have the potential to be affected by a variety of other non-inflammatory factors such as tumors, and these uncertainty biases may be decreased by the LAR (32). For another, both LDH and albumin played critical roles in inflammatory responses which was closely related to the development of pneumonia (29, 30). Moreover, albumin could also reflect the nutritional status of the patients, which was related to the complications of stroke (33). Given above, by combing the LDH and albumin, LAR can simultaneously reflect systemic inflammation and nutritional status, which may have higher value in predicting SAP. Our study have proved that the predictive value of LAR in SAP is better than LDH and albumin with 74.2% accuracy, 75.9% specificity and 63.8% sensitivity.

This study had several limitations. (1) this was a retrospective study, and we were unable to draw a causal relationship between LAR and SAP; (2) patients were recruited in a single center in China, whether the results could be applied to other regions needed further validation. (3) we only measured blood samples on admission, and we could not detect the fluctuations of LDH and albumin during hospitalization. Future studies are needed to address this issue, which will be of high value.

## Conclusion

High LAR level was associated with an increased risk of SAP in AIS patients. Patients with high LAR level (LAR > 6.75) should be alert to SAP, and more appropriate measures should be taken to reduce the risk of SAP.

## Data Availability Statement

The raw data supporting the conclusions of this article will be made available by the authors, without undue reservation.

## Ethics Statement

The studies involving human participants were reviewed and approved by Ethics Committee of the First Affiliated Hospital of Wenzhou Medical University. Written informed consent for participation was not required for this study in accordance with the national legislation and the institutional requirements.

## Author Contributions

SC and WR contributed to the conception and design of the study. QH and CD collected the clinical data. QH contributed significantly to manuscript preparation. DY, QH, and CD wrote the manuscript and performed the analysis with constructive discussions. All authors contributed to the article and approved the submitted version.

## Conflict of Interest

The authors declare that the research was conducted in the absence of any commercial or financial relationships that could be construed as a potential conflict of interest.

## Publisher's Note

All claims expressed in this article are solely those of the authors and do not necessarily represent those of their affiliated organizations, or those of the publisher, the editors and the reviewers. Any product that may be evaluated in this article, or claim that may be made by its manufacturer, is not guaranteed or endorsed by the publisher.
